# Loss of ABCB4 attenuates the caspase-dependent apoptosis regulating resistance to 5-Fu in colorectal cancer

**DOI:** 10.1042/BSR20171428

**Published:** 2018-02-21

**Authors:** Hanqing Hu, Meng Wang, Xu Guan, Ziming Yuan, Zheng Liu, Chaoxia Zou, Guiyu Wang, Xu Gao, Xishan Wang

**Affiliations:** 1 Department of Colorectal Surgery, the Second Affiliated Hospital of Harbin Medical University, Harbin, Heilongjiang, China; 2 Department of Colorectal Surgery, Cancer Institute and Hospital, Chinese Academy of Medical Sciences, Peking Union Medical College, Beijing, China; 3 Department of Biochemistry and Molecular Biology, Harbin Medical University, Harbin, Heilongjiang, China

**Keywords:** ABCB4, apoptosis, chemoresistance, colorectal cancer, 5-Fu

## Abstract

The adenosine triphosphate-binding cassette (ABC) is a large group of proteins involved in material transportation, cellular homeostasis, and closely associated with chemoresistance. ATP-binding cassette protein B4 (ABCB4) is a member of ABCs which has a similar structure to ABCB1, but fewer researches were performed. The present study is aimed to investigate the putative mechanism of ABCB4 in 5-fluorouracil (5-Fu) resistance. Then, we found that ABCB4 was significantly down-regulated in the 5-Fu resistant HCT8 cell lines by polymerase chain reaction (PCR) and Western blot. The knockdown of ABCB4 by small interfering RNA decreased the apoptosis by 5-Fu in resistant HCT8R cell lines without influencing the proliferation. Also, we found a lower expression of cleaved caspase and PARP by Western blot after the knockdown of ABCB4. However, the knockdown of ABCB4 did not influence the proliferation and apoptosis. Furthermore, the histological detection of ABCB4 mRNA level in human colorectal cancer tissues and even in the recurrent tissues after 5-Fu single-agent chemotherapy was employed to provide more concrete evidence that ABCB4 may be a tumor suppressor gene to regulate chemoresistance in colorectal cancer. Moreover, a 109-patient cohort revealed that ABCB4 predicted a poor recurrence-free survival and overall survival. In summary, ABCB4 was down-regulated in the 5-Fu resistant cells and knockdown of ABCB4 alleviated the cell apoptosis and predicts a shorter recurrence-free survival and overall survival.

## Introduction

The colorectal cancer (CRC) is a major cause of cancer-related mortality [1]. It arises from the accumulation of a serial genetic mutation in the normal epithelium, ranging from the development of adenoma to cancer [2]. The adjuvant therapeutic modalities such as radiotherapy and chemotherapy are applied to eliminate the residual cancer cells after surgery, but their efficiency is limited by the recurrence resulted from the resistance to the drugs.

The 5-fluorouracil (5-Fu) is a cornerstone of systemic chemotherapy for treatment of colorectal cancer [3,4]. It brings about the cytotoxicity by inhibiting the thymidylate synthase as well as incorporating fluoronucleotides into RNA and DNA [5]. It has been reported that some CRC patients are primarily resistant to 5-Fu based chemotherapy and some will acquire the resistance after chemotherapy. Thus, it is necessary to reveal the potential targets for treatment of CRC patients with 5-Fu resistance.

ABC transporters, a large group of protein membrane complexes, divided into seven subclasses ranging from ABC-A to ABC-G [6]. Such a superfamily plays an important role in the resistance of cancer. Multidrug resistance protein 1 (MDR1), also known as P-glycoprotein (P-gp) or ABCB1, functions as a wide-spectrum therapeutic resistant factor by drug efflux [7–9]. The ATP-binding cassette protein C2 (ABCC2) modulates the resistance to cisplatin, methotrexate, doxorubicin, mitoxantrone, and etoposide [10–13]. The other member, ABCG2, is expressed in the apical surface of proximal tubule cells such as enterocytes and hepatocytes [14,15], contributing to the distribution and elimination of many drugs [8,9].

The ABCB4 was found to locate on the canalicular membrane of hepatocytes secreting phosphatidylcholine into bile to protect the hepatobiliary epithelial against the damage from free bile acids and defects of ABCB4 cause rare biliary diseases [16–19]. ABCB4 shares similarities with ABCB1/MDR1, another member of ABC protein superfamily, in amino acid sequences [20]. Both members are polytopic transmembrane glycoproteins with two transmembrane modules each spanning the membrane six times and two ATP-binding domains [21]. However, the involvement of ABCB4 in colorectal cancer resistance remains to be illustrated.

In the present study, we found lower expression of ABCB4 in the 5-Fu resistant HCT8 colon cancer cell lines (HCT8R) than the corresponding 5-Fu sensitive HCT8 cells (HCT8S). And then, we validated a hypothesis that the loss of ABCB4 enhanced the resistance to 5-Fu via inhibiting apoptosis. Besides, ABCB4 was down-regulated in the primary tumor tissues and recurrent tissues. Furthermore, low expression of ABCB4 predicted a poor survival. ABCB4 may serve as a clinical marker in colorectal cancer patients.

## Methods

### Cell culture

Human CRC HCT8S and HCT8R were purchased from Shanghai Meixuan Corporation (Shanghai, China). HCT8S cell lines were cultured in Dulbecco’s Modified Eagle’s medium (GIBCO, Carlsbad, CA, U.S.A.) supplemented with 10% fetal bovine serum (GIBCO, Carlsbad, CA, U.S.A.). HCT8R cell lines were cultured in RMPI 1640 medium (GIBCO, Carlsbad, CA, U.S.A.) with 10% fetal bovine serum and 15 μg/ml 5-Fu (Sigma-Aldrich, Northbrook, IL, U.S.A.) according to the manufacturer’s protocols. Both kinds of cells were maintained in an atmosphere containing 5% CO_2_ at 37°C according to a previous report [22].

### Colony formation and cell proliferation assay

The 3-(4,5-dimethylthiazol-2-yl)-2,5-diphenyltetrazolium bromide (MTT) assay was performed to detect the cell proliferation according to a study previously reported [23]. Cells were plated in six-well plates at 500 cells per well in 2 ml of complete media. At the end-point, cells were stained with 0.1% Crystal Violet and number of colonies was counted.

### Flow cytometry

Apoptosis was examined by flow cytometric analysis. An Annexin V ﬂuorescein isothiocyanate/propidium iodide (FITC/PI) double stain assay (BD Biosciences, San Jose, CA, U.S.A.) was performed following the manufacturer’s protocol. The analysis was performed with FlowJo software (Treestar,Inc., San Carlos, CA, U.S.A.). All the assays were performed in triplicate.

### Western blot

Western blot was performed using standard techniques as described previously [24]. Total cell extracts were collected and quantified using BCA Protein Assay Kit (Wanlei Bio, Shenyang, China) according to the manufacturer’s protocols. Fifty micrograms of proteins were electrophoresed through 10% sodium dodecyl sulfate (SDS) polyacrylamide gels and then transferred onto polyvinylidene ﬂuoride (PVDF) membranes (Millipore, U.S.A.). The membranes were blocked with 5% non-fat milk in TBST for 2 h at room temperature and incubated with primary antibodies overnight at 4°C. Secondary antibodies labeled with HRP were used to incubate the membrane at room temperature for 2 h and the signals were detected using ECL Kit (Wanlei Bio, Shenyang, China). Subsequently, the images were analyzed by ImageJ 1.43 software. A β-actin antibody was used as a control for the whole-cell lysates. Antibodies are listed as follows: β-actin (1:1000, Cell Signaling Technology, Danvers, MA, U.S.A.), ABCB4 (1:500, ABcam, Cambridge, MA, U.S.A.), cleaved caspase-3 (1:1000, Cell Signaling Technology, Danvers, MA, U.S.A.), caspase-3 (1:1000, Cell Signaling Technology, Danvers, MA, U.S.A.), PARP (1:1000, Cell Signaling Technology, Danvers, MA, U.S.A.), cleaved PARP (1:1000, Cell Signaling Technology, Danvers, MA, U.S.A.), and anti-Rabbit or anti-Mouse HRP antibody (1:10000, Cell Signaling Technology, Danvers, MA, U.S.A.).

### RNA retraction and qRT-PCR

Total RNA was extracted from tissues and cells with TRIzol reagent (Invitrogen, Carlsbad, CA, U.S.A.) according to manufacturer’s instruction. One microgram of total RNA was reverse-transcribed using the High Capacity cDNA Reverse Transcription Kit (Applied Biosystems, Foster City, CA, U.S.A.) according to the manufacturer’s instructions. DNA was quantified using Nanodrop 2000 spectrophotometer (Thermo Fisher Scientific, Waltham, MA, U.S.A.). One microliter of DNA (0.8 ng) was used in each 20 μl of mixes performed by 40 cycles on triplicate samples in a reaction mix of SYBR Green (Thermo Fisher Scientific, Waltham, MA, U.S.A.) with Applied Biosystem 7500 quantitative PCR system (Applied Biosystems, Foster City, CA, U.S.A.) as previously described [25]. The mRNA levels were normalized against β-actin and the final data was shown as −ΔΔ*C*_t_ according to the formula: −ΔΔ*C*_t_ = −[(*C*_t, target gene_ − *C*_t, house keeping gene_)_treatment_ − (*C*_t, target gene_ − *C*_t, house keeping gene_)_nontreatment_]. Sequences of the primers are as following: ABCB4 forward primer, 5′-atagctcacggatcaggtctc-3′; ABCB4 reverse primer: 5′-ggatttagcgacaaggaaa-3′; β-actin forward primer, 5′-tggcaccagcacaatgaa-3′; β-actin reverse primer, 5′-ctaagtcatagtccgcctagaagca-3′.

### Oligonucleotide transfection

siRNA was purchased from GenePharma (Shanghai, China). Oligonucleotide transfection was performed using the Lipofectamine 2000 transfection reagent (Thermo Fisher Scientific, Waltham, MA, U.S.A.) according to manufacturer’s instructions, while nonspecific siRNA was used as negative controls. The sequences of siRNAs were presented as following. siNC, sense: 5′-uucuccgaacgugucacgutt-3′, anti-sense: 5′-acgugacacguucggagaatt-3′. siABCB4, sense: 5′-gccuauauacaaguuucautt-3′, anti-sense: 5′-augaaacuuguauauaggctt-3′.

### Ectopic expression of ABCB4 and plasmid transfection

The empty vector plasmid and ABCB4 overexpression plasmid were synthesized and purchased from Genscrpit Corporation (Nanjing, China). Both the control and the ABCB4 overexpression plasmids were transfected using the Lipofectamine 3000 transfection reagent (Thermo Fisher Scientific, Waltham, MA, U.S.A.) according to the manufacturer’s protocol.

### Patient samples

CRC tissues and corresponding normal tissues were obtained from 109 patients or 8 recurrent patients who received colectomy in the Department of Colorectal Cancer Surgery, the Second Affiliated Hospital of Harbin Medical University between March 2012 and March 2014. The samples were frozen immediately in liquid nitrogen. The recurrence was defined as progressive disease according to the Response Evaluation Criteria in Solid Tumors (RECIST) principles. All clinical samples were collected with written informed consents from patients in our department, and the ethical approval was granted from the Review Board of Hospital Ethics Committee, the Second Affiliated Hospital of Harbin Medical University.

### Statistical analysis

Statistical analyses were carried out using GraphPad Prism, version 6.0 (GraphPad, La Jolla, CA, U.S.A.) or SPSS, version 23.0 (SPSS Inc, Chicago, IL, U.S.A.). Data from at least three independent experiments performed in triplicates are presented as the means ± SD. Error bars in the scatterplots and the bar graphs represent SD. Data were examined to determine whether they were normally distributed with the One-Sample Kolmogorov–Smirnov test. If the data were normally distributed, comparisons of measurement data between two groups were performed using independent sample *t* test and the comparisons among two groups. If not normally distributed, performed by nonparametric test. The difference of the survival of between different groups was carried out using the Log-rank test or COX regression. Statistical tests were two-tailed and a *P* value of less than 0.05 was considered statistically significant.

## Results

### ABCB4 is down-regulated in HCT8R cell lines

**Figure 1:**
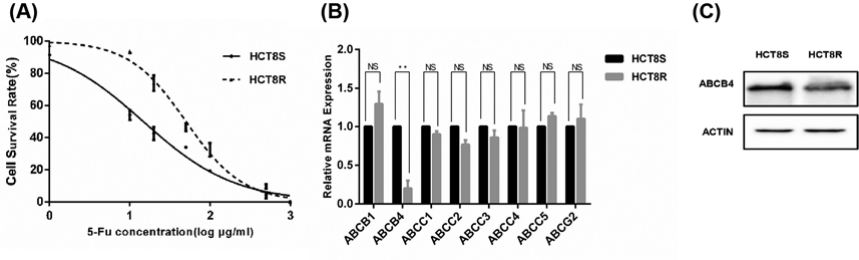
ABCB4 is down-regulated in the HCT8R cancer cells (**A**) The cytotoxic effect of 5-Fu on the HCT8S and HCT8R cells. Cell viability was measured by MTT assay after 24 h treatment with 5-Fu. Data are mean ± SD from three independent experiments. (**B**) ABCB4 was the most significantly down-regulated among other resistance-related ABC family members. Data are mean ± SD from three independent experiments; **P<0.01. (**C**) Western blot revealed that ABCB4 was down-regulated in HCT8R cells.

First of all, the resistance to 5-Fu in HCT8R was validated using MTT assay. The 50% inhibitory concentration (IC50) value was much higher in HCT8R cells compared with the HCT8S cells (50.17 ± 1.84 μg/ml vs 14.73 ± 2.64 μg/ml, *P*<0.001) (Figure 1A). In order to explore ABC members involved in the mechanism of HCT8R cells, qRT-PCR was carried out to detect the expression of ABCs. Among the genes, ABCB4 was the most notably down-regulated in the HCT8 5-Fu resistant cells (Figure 1B). Furthermore, Western blot assay verified the down-regulation of ABCB4 in HCT8R cells (Figure 1C).

### Knockdown of ABCB4 enhanced the resistance in HCT8S cells

**Figure 2:**
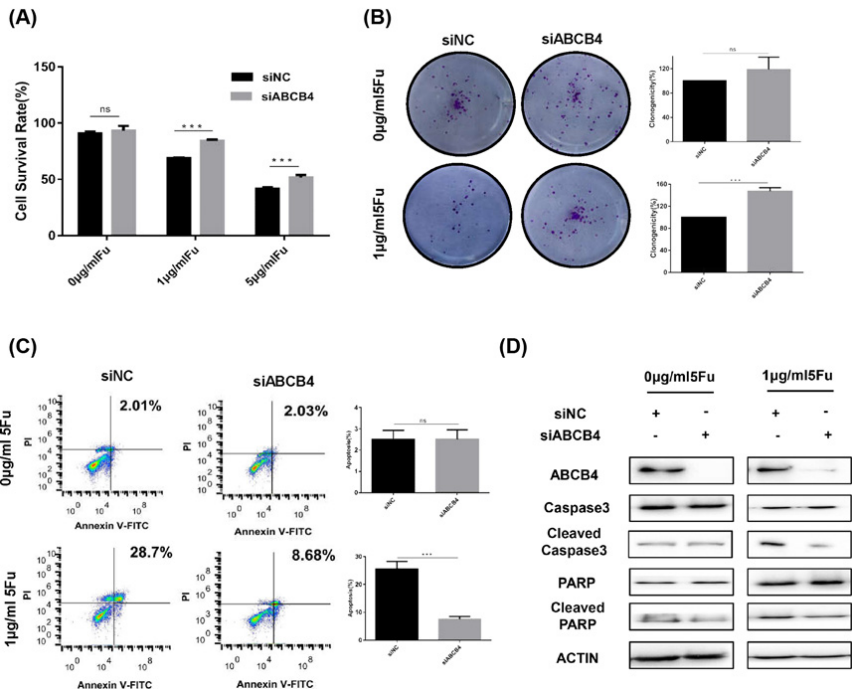
Knockdown of ABCB4 decreases apoptosis to regulate the resistance in the HCT8S cells (**A**) Knockdown of ABCB4 significantly increased the chemoresistance of HCT8S cells, but had no effect on the cell viability without 5-Fu treatment. Cell viability was detected by MTT assay. Data are mean ± SD from three independent experiments; ***P<0.001. (**B**) The colony formation assay were performed with or without the treatment of 5-Fu after the knockdown of the ABCB4. (**C**) Cell apoptosis by flow cytometry analysis after Annexin V-FITC and PI double staining. Data are mean ± SD from three independent experiments; ***P<0.001. (**D**) The knockdown of ABCB4 was confirmed by Western Blot. Apoptosis-related proteins were detected by Western Blot with or without 5-Fu treatment.

Next, to determine the function of ABCB4 in HCT8 cells, we knocked down the ABCB4 expression by siRNA and the efficacy of knockdown was confirmed by Western blot (Figure 2D). Then, the hypothesis that ABCB4 regulates the chemoresistance in HCT8 cells was testified. Compared with the negative control, ABCB4-knockdown increased the chemoresistance in HCT8S cells (Figure 2A). But, the ABCB4-knockdown showed no impact on the cell proliferation (Figure 2A). Colony formation assay showed that ABCB4 depletion did not regulate the proliferation of cells, but enhanced the resistance to 5-Fu (Figure 2B). Additionally, depletion of ABCB4 decreased the cell apoptosis only in 5-Fu treatment group by flow cytometry (Figure 2C). Consistently, Western blot assay revealed reducing cleaved caspase-3 and cleaved PARP after ABCB4 knockdown with 5-Fu treatment (Figure 2D). Above all, these results suggest that suppression of ABCB4 enhances the resistance to 5-Fu through decreasing the cell apoptosis.

### Ectopic expression of ABCB4 enhanced the sensitivity in HCT8R cells

**Figure 3:**
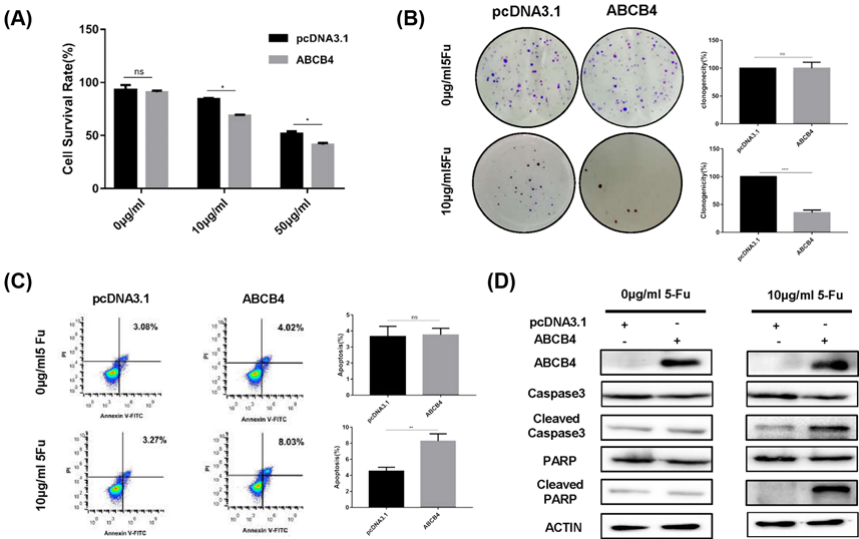
Overexpression of ABCB4 induces apoptosis to regulate the sensitivity in the HCT8R cells (**A**) Overexpression of ABCB4 significantly increased the sensitivity of HCT8R cells. Cell viability was detected by MTT assay. Data are mean ± SD from three independent experiments; *P<0.05. (**B**) The colony formation assay were performed with or without the treatment of 5-Fu after the overexpression of the ABCB4. (**C**) Cell apoptosis by flow cytometry analysis after Annexin V-FITC and PI double staining. Data are mean ± SD from three independent experiments; **P<0.01.***P<0.001. (**D**) The overexpression of ABCB4 was confirmed by Western Blot. Apoptosis-related proteins were detected by Western Blot with or without 5-Fu treatment.

To verify ABCB4 as a tumor suppressor, we ectopically expressed the ABCB4 in HCT8R cells (Figure 3D). Via MTT assay, ectopically expressed ABCB4 exhibited enhanced sensitivity to 5-Fu, but had no effect on proliferation without 5-Fu treatment (Figure 3A). Colony formation assay also confirmed that ABCB4 boosted the sensitivity to 5-Fu in HCT8R cells (Figure 3B). Besides, overexpression of ABCB4 induced the apoptosis caused by 5-Fu by flow cytometry (Figure 3C). Consistently, Western blot assay showed increasing cleaved caspase-3 and cleaved PARP caused by 5-Fu after ectopical expression of ABCB4 (Figure 3D). Taken together, these results imply that ABCB4 enhances sensitivity to 5-Fu via inducing apoptosis.

### ABCB4 was down-regulated in the colorectal cancer patients and predicted a poor survival

**Figure 4:**
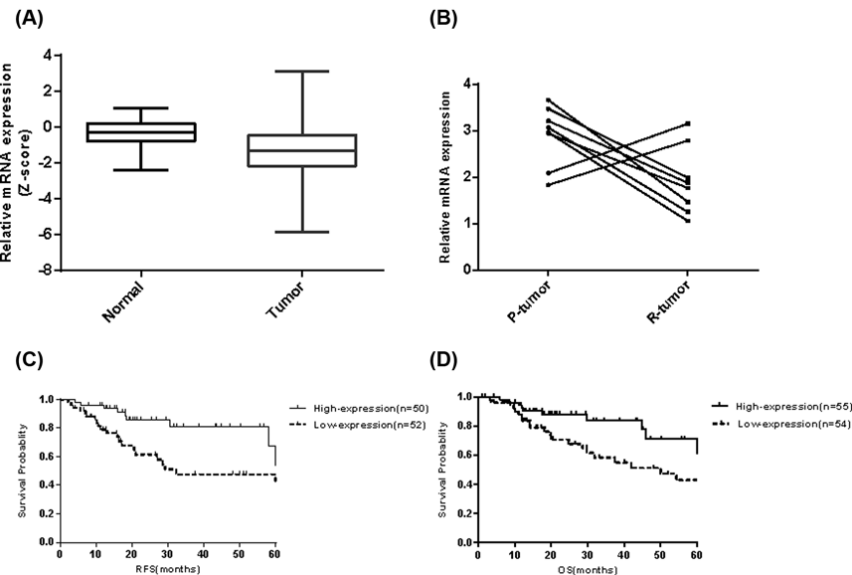
ABCB4 is significantly associated with clinical prognosis (**A**) ABCB4 was significantly down-regulated in the tumor tissues from TCGA(COADREAD) cohort; *P*<0.0001. (**B**) ABCB4 was down-regulated in the recurrent tissues compared with the first primary tumor. P-tumor represents the first primary tumor and R-tumor represents the corresponding recurrent tumor; *P*=0.039. (**C**) Kaplan–Meier curves showed that ABCB4 expression is significantly associated with a shorter recurrence-free survival in CRC patients; Log-rank *P*<0.01. (**D**) Kaplan–Meier curves showed that ABCB4 expression is significantly associated with a shorter overall survival in CRC patients; Log-rank *P*=0.03.

To investigate potential clinical significance of ABCB4, we analyzed the expression of ABCB4 in normal and tumor tissues from the clinical patients. As expected, ABCB4 was significantly decreased in the tumor tissues of TCGA(COADREAD) Cohort (Figure 4A). A 109-patient CRC cohort (Cohor1) and 8-patitnet recurrent cohort (Cohort2) was selected from our department and the demographic characteristics were shown (Tables 1 and 2). Thus, we examined the expression of ABCB4 in eight recurrent patients after 5-Fu based single-agent chemotherapy by qRT-PCR. Compared with the p-tumor tissues, ABCB4 was significantly lower in the recurrent tumor tissues (Figure 4B). Next, we examined the putative clinical significance of ABCB4 in our cohort. Lower expression of ABCB4 predicted a shorter RFS (Figure 4C) and OS (Figure 4D). Univariate and multivariate COX regression analyses revealed that the expression of ABCB4 was an independent predictor of CRC aggressiveness (Table 3). Our data indicate that ABCB4 is pathologically and clinically associated with CRC recurrence and outcomes.

**Table 1:** Demographic characteristics of the patients in Cohort1

Variables	High-expression (*n*=55)	Low-expression (*n*=54)	*P*
Age	62.35 ± 14.32	62.91 ± 13.55	0.834
Gender			0.389
Male	30(54.5%)	25(46.3%)	
Female	25(45.5%)	29(53.7%)	
TNM Stage			0.367
Early stage(stage I + stage II)	31(56.4%)	35(64.8%)	
Late stage(stage III + stage IV)	24(43.6%)	19(35.2%)	
Perineural invasion			0.19
YES	24(43.6%)	17(31.5%)	
NO	31(56.4%)	37(68.5%)	
Lymphatic invasion			0.884
YES	17(30.9%)	16(29.6%)	
NO	38(69.1%)	38(70.4%)	
Venous invasion			0.254
YES	19(34.5%)	13(24.5%)	
NO	36(65.5%)	41(75.5%)	

**Table 2:** Demographic characteristics of the patients in Cohort2

	Age	Gender	TNM Stage	Primary tumor site	Perineural invasion	Lymphatic invasion	Venous invasion	regime	RFS (months)
Case1	56	Male	II	Ascending colon	No	No	No	Capecitabine (single)	37.5
Case2	61	Male	II	Ascending colon	No	No	No	Capecitabine (single)	26.8
Case3	57	Male	II	Ascending colon	No	No	No	Capecitabine (single)	41.3
Case4	58	Female	II	Sigmoid colon	No	No	No	Capecitabine (single)	28.7
Case5	55	Male	II	Ascending colon	No	No	No	Capecitabine (single)	45.1
Case6	55	Male	II	Rectum	No	No	No	Capecitabine (single)	33.8
Case7	52	Female	II	Rectum	No	No	No	Capecitabine (single)	32.7
Case8	69	Male	II	Rectum	No	No	No	Capecitabine (single)	35.6

**Table 3:** Univariate and multivariate analysis of recurrence-related factors in CRC patients

Variable	Univariate		Multivariate	
	Hazard ratio (HR, 95%CI)	*P*	HR (95%CI)	*P*
Age	0.998 (0.972–1.024)	0.86	0.997 (0.966–1.028)	0.840
Gender				
Male	2.654 (1.273–5.534)	0.007	3.605 (1.552–8.374)	0.003
Female	1		1	
TNM stage				
Early stage (stage I + stage II)	0.481 (0.241 to 0.959)	0.038	0.384 (0.173–0.852)	0.019
Late stage (stage III + stage IV)	1		1	
Perineural invasion				
YES	1		1	
NO	0.824 (0.408–1.664)	0.590	1.180 (0.524–2.660)	0.689
Lymphatic invasion				
YES	1		1	
NO	0.480 (0.237 to 0.973)	0.042	0.819 (0.292–2.300)	0.705
Venous invasion				
YES	1		1	
NO	0.564 (0.275–1.158)	0.119	0.888 (0.317–2.488)	0.821
ABCB4 expression				
High	1		1	
Low	2.142 (1.032–4.449)	0.041	2.743 (1.291–5.827)	0.01

## Discussion

The potential roles of ABC family members involved in the chemoresistance have been reported extensively [8–15]. Increasing evidences have shown ABCB4 plays a part in the cancer resistance and growth. A microarray-based study has shown that ABCB4 modulates the doxorubicin resistance in ovarian cells [26] and the paclitaxel resistance in breast cancer cells [27]. Furthermore, caused by the hypermethylation of CpG promoter, ABCB4 is epigenetically silenced to initiate the tumor growth [28]. In the pediatric medulloblastoma, high expression of ABCB4 links to the radiation resistance [29]. Inspite of some controversies, ABCB4 is worthy of consideration as a target for illustrating the resistance in human cancers. In addition to the molecular researches, a population-based research showed lower transcript level of ABCB4 in accordance with a shorter disease-free interval in colorectal cancer patients treated by adjuvant chemotherapy [30]. However, the question of whether ABCB4 functions in CRC has not yet been explored.

In the present study, the relationship between ABCB4 and chemoresistance has been established and the insights of mechanism to implication of apoptosis induced by ABCB4 with 5-Fu treatment have also been described. We show that ABCB4 is down-regulated at the mRNA and protein level in the HCT8R cells. Knockdown of ABCB4 suppresses the caspase-dependent apoptosis pathway, which is canonical in regulating the resistance in cancer [31–33]. In order to verify the results, overexpression of ABCB4 in HCT8R cells has been performed. We have found that overexpression of the ABCB4 enhanced the sensitivity to 5-Fu and induced the apoptotic response in the HCT8R cells. All these data have shown that ABCB4 regulates the chemoresistance to 5-Fu in the HCT8 cells. Our data have some similarities with the published papers. A previous study provided evidence that loss of ABCB4 enhances the cell proliferation in many cancers [28]. In addition to the biological importance, we found that ABCB4 is down-regulated in the CRC and recurrent patients. Moreover, ABCB4 has an association with the risk of recurrence and overall survival, which is in accordance with a previous report [30]. Our works suggest that ABCB4 may serve as a predictor for recurrence after 5-Fu based chemotherapy in CRC patients. Thus, it may be important to classified the patients according to the ABCB4 expression to provide a more precious therapeutic strategy.

Although ABCB4 shares some similarity with ABCB1 [20], the ABCB4 showed a different pattern in the colorectal cancer. Inspite of high sequence identity with the ABCB1, ABCB4 had a transcript of 4100 nucleotides, 400 nucleotides less than the transcript of ABCB1 [20]. The N-terminal domain of ABCB4, different form other region, is not conserved with the ABCB1 and this region is important for the function of ABCB4, which had shown association with the LPAC syndrome and ICP [34]. The N-terminal of ABCB4 also has a phosphorylated site, which was involved in the substrate transportation [34]. As for the function in cancer, there some ABC members reported to be down-regulated such as ABCA7, ABCA12, ABCB2, ABCB5, and ABCD1 in the melanoma [35]. This suggests that pattern of lower expression in ABC family members may play a part in the cancer. ABCB4 has been found to be inactivated in many epithelial cancers including the lung, breast, and head and neck, which is due to the hypermethylation of the promoter [36–39]. The direct evidence have shown that loss of ABCB4 promotes the proliferation in the lung cancer [28]. These previous researches support the hypothesis that ABCB4 may function in cancers via inactivation.

We do acknowledge some limitations of our study. For instance, only one cell line is included, which cannot print a whole picture of cancer due to the heterogeneity and lack of the influence from the circumferential environments without *in vivo* assay. To set off these drawbacks, we validate the results acquired from cell line in three patient cohorts. Just as we planned, consistent outcomes have been observed in these cohorts.

In summary, we have redicated a novel association of ABCB4 and chemoresistance in CRC. Our findings provide insights into the mechanism of ABCB4, as we have identified that ABCB4 induce apoptosis in CRC. Most of all, ABCB4 may act as a valuable clinical factor predicting the survival in CRC. Further investigations could gear toward understanding the effects of ABCB4 on chemoresistance *in vivo*.

## Abbreviations

5-Fu: 5-fluorouracil
ABC: adenosine triphosphate-binding cassette
ABCB1: ATP-binding cassette protein B1
ABCC2: ATP-binding cassette protein C2
ABCG2: ATP-binding cassette protein G2
COX: proportional hazards model
CRC: colorectal cancer
FITC: fluorescein isothiocyanate
HCT8: human colon cancer cell line
HCT8R: HCT8 5-Fu resistant cell line
HCT8S: HCT8 5-Fu sensitive cell line
HR: hazard ratio
HRP: horseradish peroxidase
IC50: 50% inhibitory concentration
ICP: Intrahepatic cholestasis of pregnancy
LPAC: low phospholipid associated cholelithiasis
MDR1: multidrug resistance protein 1
MTT: 3-(4,5-dimethylthiazol-2-yl)-2,5-diphenyltetrazolium bromide
NC: negative control
PAGE: polyacrylamide gel electrophoresis
PCR: polymerase chain reaction
PARP: poly ADP-ribose polymerase
P-gp: P-glycoprotein
PI: propidium iodide
PVDF: polyvinylidene fluoride
qRT-PCR: quantitative reverse-transcription polymerase chain reaction
RECIST: Response Evaluation Criteria in Solid Tumors
RFS: recurrence-free survival
SDS: sodium dodecyl sulfate
siRNA: small interfering RNA
TCGA: the cancer genome atlas
TNM: TNM staging system
